# Multifunctionality of Calebin A in inflammation, chronic diseases and cancer

**DOI:** 10.3389/fonc.2022.962066

**Published:** 2022-09-16

**Authors:** Aranka Brockmueller, Anna-Lena Mueller, Ajaikumar B. Kunnumakkara, Bharat B. Aggarwal, Mehdi Shakibaei

**Affiliations:** ^1^ Musculoskeletal Research Group and Tumor Biology, Chair of Vegetative Anatomy, Faculty of Medicine, Institute of Anatomy, Ludwig-Maximilians-University Munich, Munich, Germany; ^2^ Cancer Biology Laboratory and DBT-AIST International Center for Translational and Environmental Research (DAICENTER), Department of Biosciences and Bioengineering, Indian Institute of Technology (IIT) Guwahati, Guwahati, India; ^3^ Inflammation Research Center, San Diego, CA, United States

**Keywords:** Calebin A, turmeric, NF-κB, mitogen-activated protein kinase (MAPK), chronic inflammation, signaling pathways, tumor prevention, cancer treatment

## Abstract

Chronic diseases including cancer have high case numbers as well as mortality rates. The efficient treatment of chronic diseases is a major ongoing medical challenge worldwide, because of their complexity and many inflammatory pathways such as JNK, p38/MAPK, MEK/ERK, JAK/STAT3, PI3K and NF-κB among others being implicated in their pathogenesis. Together with the versatility of chronic disease classical mono-target therapies are often insufficient. Therefore, the anti-inflammatory as well as anti-cancer capacities of polyphenols are currently investigated to complement and improve the effect of classical anti-inflammatory drugs, chemotherapeutic agents or to overcome drug resistance of cancer cells. Currently, research on Calebin A, a polyphenolic component of turmeric (*Curcuma longa*), is becoming of growing interest with regard to novel treatment strategies and has already been shown health-promoting as well as anti-tumor properties, including anti-oxidative and anti-inflammatory effects, in diverse cancer cells. Within this review, we describe already known anti-inflammatory activities of Calebin A *via* modulation of NF-κB and its associated signaling pathways, linked with TNF-α, TNF-β and COX-2 and further summarize Calebin A’s tumor-inhibiting properties that are known up to date such as reduction of cancer cell viability, proliferation as well as metastasis. We also shed light on possible future prospects of Calebin A as an anti-cancer agent.

## 1 Introduction

Current statistics show that at least 18 million of people worldwide are newly diagnosed with cancer every year. Approximately half of all cancer cases in men are composed of lung (LC), prostate, colorectal (CRC), stomach and liver cancer and these cancers account for more than half of all cancer-related deaths in men. In women, breast cancer (BC) is the leading cause of cancer incidences with about a quarter of new cases every year. Colorectal, lung and cervix uteri cancer account together for another quarter of new cancer cases. Combined, these four diseases represent about 45% of the cancer mortality rate in women (1). Many of patients suffering from cancer are treated with drugs, mostly with classical chemotherapeutic agents. As a result of these mono-targeting strategy and due to long treatment durations, cancer cells can become less receptive to the administered drugs or even develop resistance (2, 3), often leading to loss of control of the cancer disease. Thereby, inflammation and inflammatory processes are considered as the key drivers of health problems in many diseases, including chronic disorders such as rheumatoid arthritis (4), bowel diseases (5) as well as cancer (6). In general, acute inflammations can become chronic, consequently resulting in cell degeneration (7) development. In addition, self-accelerating, inflammation-propagating and cancer-promoting events are taking place within cancer cells that can be triggered *via* various signaling pathways such as tumor necrosis factor (TNF)-α- or -β-induced cascades that promote pro-inflammatory master transcription factor nuclear factor kappa-light-chain-enhancer of activated B-cells (NF-κB) expression and its related gene end products, finally peaking in an tumor environment with improved vitality, proliferation, migration and invasion properties of cancer cells (8). For this reason, novel approaches and multi-modulatory treatment strategies and improvements are constantly being sought, whereas secondary plant compounds play an important role.

Polyphenols, such as curcumin (derived from *Curcuma longa*) and resveratrol (derived from *Vitis vinifera*), are already well researched plant-derived compounds that have proven multiple health-promoting properties including the protection of cardiovascular organs (9, 10) and nerves (11, 12). Their modulatory interventions in signaling pathways of inflammation are of particular note, as cancer pathogenesis is mostly preceded or underpinned by inflammatory events (6, 13). Furthermore, even many direct anti-cancer effects such as inhibition of cell vitality, proliferation and metastasis (14) have been demonstrated.

Now, Calebin A, a new compound isolated from the spice and medicinal *Curcuma* species plants (15) is becoming increasingly interesting in a scientific context. It has already shown versatile application potential in the prevention and treatment of neurodegenerative diseases, metabolic disorders, diseases of the musculo-skeletal system (16–19) and presumably also infectious diseases. Calebin A’s ability to target inflammatory cascades, especially the major pro-inflammatory transcription factor NF-κB and associated signaling pathways, might be of critical importance. Thus, Calebin A could not only be used as a modulator in numerous inflammatory diseases, but also became relevant in the prevention and co-treatment of cancer. Therefore, we dedicate a review to Calebin A as a promising polyphenol, describing its characteristics and anti-inflammatory effects that are known up to date, including the targeted signaling cascades and its promising potential in the modulation of different cancer types.

## 2 Effects of inflammatory proteins in chronic diseases including cancer

Today, it is widely known that the majority of chronic diseases is caused by permanent inflammation within the human body, comprising tissue-related or organ-specific (e. g. neurological, pulmonal, cardiovascular) disorders as well as inflammatory bowel diseases and cancer, to name just a few (4, 20–23).

**Figure 1:**
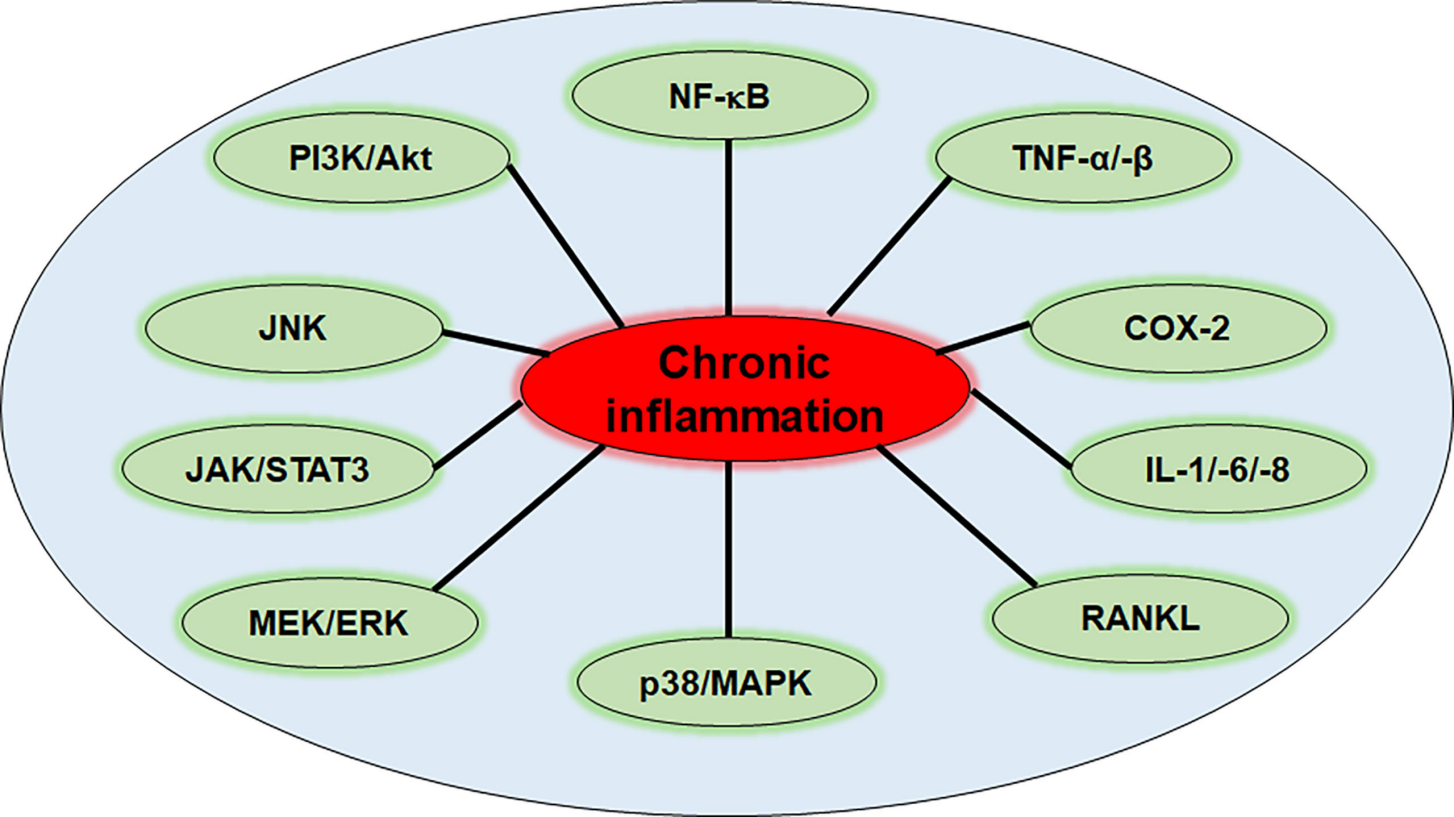
Inflammation-related pathways in chronic diseases. NF-κB, nuclear factor kappa-light-chain-enhancer of activated B-cells; TNF-α/-β, tumor necrosis factor α/β; COX-2, cyclooxygenase-2; IL-1/-6/-8, interleukin-1/-6/-8; RANKL, receptor activator of NF-κB ligand; p38/MAPK, protein kinase of 38 kDa/mitogen-activated protein kinase; MEK/ERK, mitogen-activated protein kinase kinase/extracellular signal-regulated kinase; JAK/STAT3, Janus kinase/signal transducer and activator of transcription 3; JNK, c-Jun N-terminal kinase; PI3K/Akt, phosphoinositide 3-kinase/serine-threonine protein kinase B.

These inflammation-associated chronic diseases mostly come along with pain, severe restrictions of patient’s daily lives, long periods of suffering due to difficult diagnosis and mono-therapy often holding undesirable side effects in long-term treatment administration (24, 25). Although the range of chronic diseases is enormous, targeting the underlying trigger that they all have in common, namely inflammation, represents a powerful strategy in their treatment (26–30). However, better knowledge and deeper understanding of different inflammatory mechanisms and signaling pathways is mandatory in order to develop sustainable treatment approaches. Up to date, major pathways and mediators contributing to inflammation and inflammation-associated processes in chronic diseases, especially in cancer, are known, whereat NF-κB, mitogen-activated protein kinase (MAPK), c-Jun N-terminal kinase (JNK), signal transducer and activator of transcription (STAT)3, mitogen-activated protein kinase kinase/extracellular signal-regulated kinase (MEK/ERK) and phosphoinositide 3-kinase/serine-threonine protein kinase B (PI3K/Akt) signaling (**Figure 1**) play an important role among some others and will be described in more detail in the following sections.

### 2.1 Chronic diseases and the regulation of JNK

The JNKs comprise its three members JNK1, JNK2 and JNK3, and represent a subfamily of MAPK. The JNK pathway is one of the main signaling cascades of cell fate regulation since it mediates a variety of cellular processes such as apoptosis, survival and proliferation, inflammatory response and metabolism. Therefore, dysregulation of the JNK pathway plays a major role in inflammation and inflammatory processes (**Figure 1**), thus is often linked with several chronic immune disorders (31, 32). Especially in cancer and tumorigenesis, the role of JNK pathway is supported by increased levels of JNK that were found in different cancer types (33, 34). JNK can be stimulated by different stimuli including oxidative stress or deoxyribonucleic acid (DNA) damage, ultraviolet light, cytokines and pathogens activating signaling pathways downstream of different cell receptors such as G-protein coupled receptors (GPCRs), transforming growth factor-β receptors, tumor necrosis factor receptors (TNF-Rs) or Toll-like-receptors (TLRs). These pathways rely on the serial activation by phosphorylation of MAPK kinase kinases (MAP3Ks) and MAPK kinases (MAP2Ks/MKKs) known as MKK4 and MKK7, which in turn finally activate JNKs (35). The primary target of JNKs, which are considered the effectors of the JNK signaling pathway, is c-Jun and activating transcription factor (ATF)-2 activation, as components of the adaptor protein (AP)-1 complex. By activation of AP-1 many genes involved in the inflammatory response are transcribed, such as cytokines, chemokines or leukocyte adhesion molecules (36). Moreover, c-Jun activation can promote expression of key regulators of the cell cycle such as cyclin D1, which stimulates cell cycle progression or can inhibit pro-apoptotic mediators such as tumor suppressor p53, thus giving space to cancer development (37, 38).

### 2.2 Chronic diseases and the regulation of p38/MAPK

The p38/MAPK pathway represents another downstream cascade of a MAPK subfamily triggered by external stress-related stimuli such as TNF-α or interleukin (IL)-1β binding to different receptors and mediating a wide range of cellular responses (39). Like all MAPK pathway cascades, p38 signaling comprises three stages of protein kinases, including upstream MAP3Ks to downstream MAP2Ks and finally MAPKs, latter are represented by the p38 kinase family here that act as the final cascade effectors (39).

Activated p38 regulates various pro-inflammatory cytokines (**Figure 1**) including TNF-α and various ILs as well as inflammation-associated enzymes such as cyclooxygenase (COX)-2 or matrix metalloproteinases (MMPs), that are essential regulators of cell growth and survival, thus possess notable pro-tumorigenic potential (39, 40). Moreover, p38 has also an influence on the receptor activator of NF-κB ligand (RANKL), that has been found to play a crucial role in cancer development and metastasis (41, 42). Because of the close linkage of inflammatory responses and cancer development, the p38/MAPK signaling pathway (**Figure 1**) represents an important target in cancer therapy and has already been shown to act as a major determinant of therapeutic efficacy of 5-fluorouracil (5-FU), cisplatin, and radiotherapy (43, 44).

### 2.3 Chronic diseases and the regulation of MEK/ERK

The MEK/ERK signaling is one of the most well studied pathways of the MAPK cascades that is implicated in cell proliferation, migration, differentiation and survival. It is known that alterations in MAPK signaling cascades can contribute to cancer development and other chronic disease (**Figure 1**), thus targeting the ERK/MAPK rep-resents an attractive treatment and intervention strategy (45–47). ERK1/2 are serine-threonine-selective protein kinases that are activated through upstream MAPK/ERK kinases MEK1/2. Phosphorylated, thus activated ERK1/2 then translocates into the nucleus, where it initiates the transcription of numerous genes and transcription factors such as c-Fos, c-Jun, Elk1 or ATF-2, which directly control various cellular mechanisms linked with proliferation and differentiation (48, 49).

The RAS/RAF/MEK/ERK signaling pathway is activated by different growth factors and cytokines that act through receptor tyrosine kinases (RTKs), GPCR or cytokine receptors, which then activate signaling *via* activation of RAS that in turn recruits RAF, which functions as a MAP3K, inducing MEK1/2 phosphorylation (50, 51). The abnormal activation of RTK signaling or mutations in RAS or RAF genes are highly prevalent in human cancers and are assumed to act as key drivers (49, 52). RAS for example, has been found to be mutated in around 30% of all human cancers with KRAS mutation representing the most predominantly mutated isoform, in 90% of pancreatic and in more than 30% of colon cancer (50, 53–55). BRAF gene has been found to be among the most frequently mutated kinases in human cancer, too. Especially in melanoma, around 50% of tumors were observed carrying BRAF mutations (56).

### 2.4 Chronic diseases and the regulation of JAK/STAT3

The Janus kinases (JAKs), which are non-receptor cytoplasmic tyrosine kinases, belong to the main promoters of STAT activation, whereas especially STAT3 and STAT5 of the total number of seven STAT-protein family members have been observed to be of greatest relevance in cancer development. Especially signaling by the JAK/STAT3 pathway cascade (**Figure 1**) was found to majorly contribute to carcinogenesis by impacting tumor cells as well as the tumor microenvironment that is essential for cancer progression (57). Cytokines such as IL-6 are known triggers for JAK activation, initiating further cascades. In addition, also GPCRs and TLRs can induce JAK/STAT3 signaling by ligands binding to them, although it is not traditionally associated with them in first line (57). Activated JAK phosphorylates STAT3 protein then, which consequently transfers into the nucleus for gene transcription. STAT3’s target genes are mainly implicated in processes of cell differentiation, such as survivin (BIRC5), B-cell lymphoma (Bcl)-2, cyclin D1, c-Myc or vascular endothelial growth factor (VEGF), thus their expression plays a key role in cancer promotion (57–61). Moreover, STAT3’s its abnormal activation together with altered IL-6 levels are often found in chronic inflammatory diseases (**Figure 1**) as well as in the majority of patients suffering from cancer, making STAT3 and its pathway another attractive target in cancer therapy (57, 62, 63).

### 2.5 Chronic diseases and the regulation of NF-κB

NF-κB is known to strongly contribute to inflammatory processes, immune responses and cellular fate. Therefore, the NF-κB pathway that is tightly controlled under physiological conditions, has been shown to be involved in a great range of chronic diseases (**Figure 1**) such as cancer and other inflammatory immune diseases when being dysregulated (64). As NF-κB acts as a key player in the expression of several genes that are partly involved in cell proliferation and migration, it is obvious that its mis-regulation can trigger uncontrolled cell proliferation, thus tumorigenesis (64).

NF-κB is predominantly found as a heterodimer consisting of p50 and p65 resting in the cytoplasm inhibited by its inhibitor nuclear factor of kappa light polypeptide gene enhancer in B-cells inhibitor (IκB)α, thus it is transcriptionally inactive (65). When IκBα becomes phosphorylated by the IκB kinase (IKK) though, it undergoes degradation, consequently leading to NF-κB release and its nuclear. Within the nucleus, NF-κB acts as a transcription factor promoting expression of several genes that are strongly involved in immune responses and inflammation (e.g. TNF-α, TNF-β, IL1-β, MMPs, COX-2, RANKL, caspase-3, PARP), which then again act as triggers of NF-κB activation among many other stimuli (e.g. growth factors, radiation, reactive oxygen species) contributing to the establishment of a recurring circuit (64–66). This mode of action described belongs to the so-called canonical or classical pathway of NF-κB. Apart from that, NF-κB can also be activated by the non-canonical pathway that especially initiates the activation of p52/RelB by phosphorylating and processing p100 NF-κB members *via* the NF-κB-inducing kinase, which mainly integrates signals from TNF receptors that are targeted by their specific ligands. With downstream p52/RelB activation the complex translocates into the cell nucleus where transcription of genes implicated in inflammatory processes takes place (65, 67). It is known that permanent inflammation comes along with the risk of cancer development, so that prophylaxis and interruption of inflammatory cascades, like targeting NF-κB signaling (**Figure 1**), are essential in cancer therapy, since genes regulated by NF-κB have been already shown to be involved carcinogenesis (68, 69).

### 2.6 Chronic diseases and the regulation of PI3K/Akt

The PI3K/Akt pathway represents another signaling cascade that mediates important inflammatory processes as well as leukocyte function, which are crucial for generating an inflammatory environment (**Figure 1**). Thus, alterations of this pathway have frequently been found in inflammation-related disorders and in human cancers.

The class I of PI3Ks that comprises four isoforms (PI3Kα, PI3Kβ, PI3Kδ and PI3Kγ) consists of lipid-signaling kinases, which induce the phosphorylation of phosphatidylinositol 4,5-bisphosphate for phosphatidylinositol 3,4,5-trisphosphate (PIP3) synthesis. PIP3 is a second messenger that is involved in various processes of cell fate (70). The activation of Akt is a prominent example of an effector molecule of PIP3 that in turn stimulates further downstream targets including the mammalian target of rapamycin (mTOR) that regulates several processes such as cell proliferation and survival, protein synthesis, metabolism, autophagy and angiogenesis. Dysregulated PI3K/Akt/mTOR signaling has been found to trigger various hallmarks of cancer by its increased activation that has been observed. Therefore, this pathway is assumed as another potential target in the therapy and treatment of different types of cancer (71–73). In addition, also before described NF-κB can be induced by PI3K/Akt signaling leading to cytokine secretion and further triggering of other inflammation-linked pathways, finally resulting in permanent inflammation (**Figure 1**), thus increased risk of carcinogenesis (74).

With demonstrating these pathways, the important role of inflammation in chronic disease and cancer becomes obvious and the same goes for targeted treatment in their therapy. Standard treatment in cancer therapy and other inflammation-related diseases often reach their limits due to insufficient efficiency, severe side effects or development of resistances to the drugs of treatment (75–77). Therefore, in the last decades plant metabolites, that have been used over centuries in traditional medicine, gained more and more attention as co-treatment strategy because of their anti-inflammatory and immuno-modulating potential that has been shown in many studies *in vitro* as well as *in vivo* (78–82). For example, resveratrol and curcumin as quite famous polyphenols, have been demonstrated to inhibit proliferation and metastasis of cancer cells and to act as pro-apoptotic agents in cancer (76, 83, 84). In contrast, current research on Calebin A that represents another bioactive agent for fighting inflammation and associated cellular processes, is still in its infancy, although first studies showed promising results (85, 86). Thus, in the following passage Calebin A will be further described.

## 3 Properties and versatile possibilities of Calebin A

### 3.1 Origin and characteristics of Calebin A

Calebin A was first isolated from *Curcuma longa* (87) and then detected in *Curcuma caesia* (15) too. Both *Curcuma* species are Asian medicinal herbs, belonging to *Zingiberaceae* family, and their rhizomes are processed to turmeric powder, which was used against gastric, hepatic, dental or inflammatory disorders (88) in Ayurvedic medicine thousands of years before the individual components were deciphered. Now, it is a sought-after ingredient in kitchens around the world as well as a more frequently used ingredient in the cosmetic industry. Moreover, numerous research achievements enable an ever-increasing importance in modern medicine, both as prophylaxis against oxidative stress (89), inflammation (90) and as co-treatment in the context of chronic inflammatory or cancerous (91, 92) diseases.

**Figure 2:**
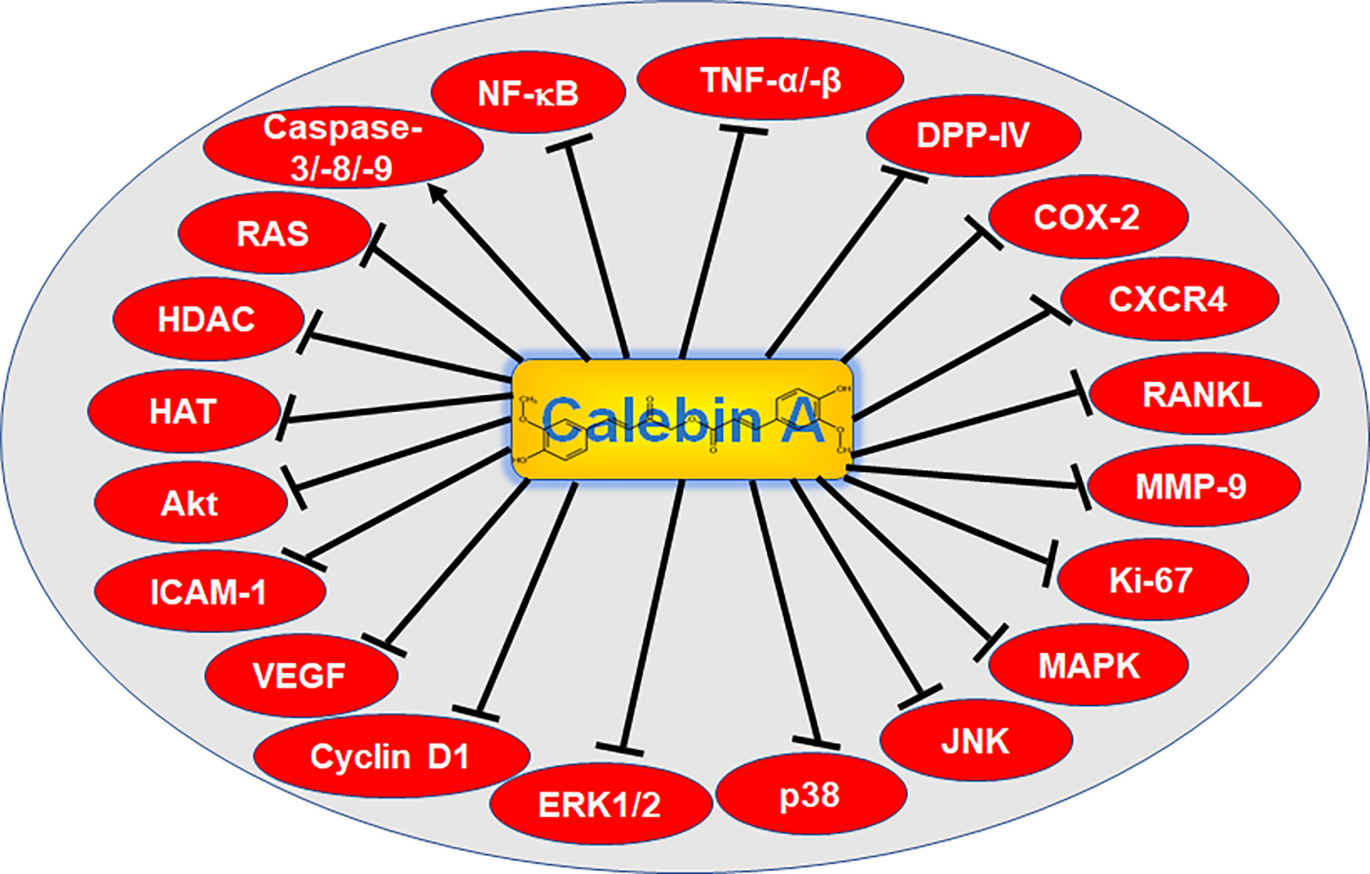
Signaling targets of Calebin A. NF-κB, nuclear factor kappa-light-chain-enhancer of activated B-cells; TNF-α/-β, tumor necrosis factor α/β; DPP-IV, di-peptidyl peptidase IV; COX-2, cyclooxygenase-2; CXCR4, C-X-C chemokine receptor type 4; RANKL, receptor activator of NF-κB ligand; MMP-9, matrix metalloproteinase-9; Ki-67, protein Ki-67; MAPK, mitogen-activated protein kinase; JNK, c-Jun N-terminal kinase; p38, protein kinase of 38 kDa; ERK 1/2, extracellular signal-regulated kinase; VEGF, vascular endothelial growth factor; ICAM-1, intercellular adhesion molecule-1; Akt, serine-threonine protein kinase B; HAT, histone acetyltransferase; HDAC, histone deacetylase; RAS, rat sarcoma.

Calebin A was chemically identified as 4’’- (3’’’- methoxy- 4’’’- hydroxyphenyl)- 2’’- oxo- 3’’- enebutanyl 3- (3’- methoxy- 4’hydroxyphenyl)propenoate, with C21H20O7 as molecular formula (18) and its polyphenolic structural formula is presented in **Figure 2**. An extensive *in vivo* study ensured the safety of this secondary phytochemical, even with the highest dose selected, 100 mg/kg/d did not elicit any signs of toxicity in female or male Wistar rats when administered orally for 90 days. It was taken care to ensure that the Calebin A-treated rats did not show any statistically significant changes in their body, organ or tissue constitution during the course of the experiments and that their metabolic parameters in the blood serum (93) remained stable. Pharmacokinetic analyses of serum and urine samples from male Sprague-Dawley rats further revealed Calebin A as an aglycone, glucuronidated metabolite with a bioavailability about 0.5%, a serum half-life of about 1-3 hours, and primarily non-renal excretion (89). Overall, Calebin A is considered a safe plant-derived substance that is suitable for use in *in vitro* as well as *in vivo* studies.

### 3.2 Health-promoting potential of Calebin A

Shortly after primary isolation of Calebin A, first *in vitro* experiments were con-ducted where it was shown that Calebin A was able to protect PC12 rat pheochromocytoma cells from β-amyloid impact (18), relevant in Alzheimer’s disease. This was in agreement with an earlier conducted study using a methanol extract of turmeric (94), although the study of phytochemicals in Alzheimer’s disease had received little attention until then. However, it was an important first step, since extracellular plaque deposition of β-amyloid is one of the main characteristics of Alzheimer’s disease pathology, which belongs to the common progressive cognitive dysfunctions. Thereafter, this protective effect could be confirmed in another study with the new finding that a concentration of 25 µg/ml of Calebin A was not harmful to cells (87), so that further research approaches can be pursued.

Moreover, there are new findings in the field of musculoskeletal diseases. For example, in canine tenocytes, Calebin A was shown to suppress inflammatory processes as well as the degradation of extracellular matrix, suggesting Calebin A as appropriate potential compound for prevention and treatment of tendinitis (17). In addition, Calebin A was found to down-regulate osteoclast formation in both RANKL- and cancer-induced differentiation of mouse macrophages into osteoclasts and thus bone loss, resulting in Calebin A acting at an important early stage of the osteoclastogenic pathway. Moreover, the suppression of osteoclast formation by Calebin A was shown to be mediated by attenuation of the RANKL signaling pathway (19).

Furthermore, Calebin A is assumed to act as multi-modulatory agent in other disorders such as obesity and non-alcoholic fatty liver diseases based on the finding that Calebin A has been observed to be non-toxic to adipocytes but simultaneously suppressed adipogenesis through inhibition of adipocyte differentiation at an early stage and down-regulation of differentiation-related factors fatty acid synthase (FAS), peroxisome proliferator-activated receptor γ (PPARγ), CCAAT/enhancer-binding protein (C/EBP)α and C/EBPβ in adipocytes. Simultaneously, AMP-activated protein kinase (AMPK) activity was enabled by Calebin A and furthermore, Calebin A was found to induce lipolysis at a concentration of 20 µM throughout the extensive research study. In addition, Calebin A even protected against obesity and reduced hepatic stenosis as a secondary disease, in high-fat diet fed C57BL/6J mice (95). Another study related to the recently demonstrated ability of Calebin A to reduce blood sugar and weight of C57BL/6J mice despite high-fat diet was conducted. Here, Calebin A acted in an anti-obese way as well and modulated thermogenesis as well as gut microbiota by enhancement of intestinal commensal bacteria such as *Akkermansia* and *Butyricicoccus* (96). Calebin A has been shown to also intervene in the intestinal metabolism by acting as a natural dipeptidyl peptidase IV (DPP-IV) inhibitor and thus delaying the break-down of the intestinal hormone GLP-1 (16). This represents an attractive treatment strategy for lowering blood sugar levels in patients with diabetes type 2. As the maintenance of the intestinal microbiome balance is fundamental for human health, Calebin A could become of great relevance in the future.

Most of the diseases mentioned above are caused by permanent inflammatory processes. Various pro-inflammatory signaling pathways that can be affected by Calebin A and its health-beneficial properties (**Figure 2**) and the far-reaching consequences of inflammation, such as cancer development and progression, will be discussed in more detail in the following chapters.

## 4 Multifunctional regulation of inflammation and cancer cascades by Calebin A

### 4.1 Anti-inflammatory activities of Calebin A

Anti-inflammatory effects of turmeric were demonstrated in 1971 for the very first time (97), while the signaling pathways that are used by Calebin A in order to exert its anti-inflammatory properties have been studied in more detail only from 2013 on (88). Despite the still early stage of research, Calebin A has already been shown to modulate several signaling pathways whose common link is represented by the major inflammatory transcription factor NF-κB (**Table 1**), which is a key player in chronic diseases and oncogenesis (64).

**Table 1:** Calebin A exhibits anti-inflammatory activities.

Pathway	Anti-inflammatory impact	Terms of study		Reference
COX-2**/**NF-κB	Calebin A blocked approximately equally COX-1 and COX-2.	*in vitro*	COX inhibitor **screening**	(89, 98, 99)
TNF-α**/**NF-κB	Calebin A inhibited TNF-α-induced NF-κB activation and IκBα degradation by suppressing DNA binding in cancer cells.	*in vitro*	KBM-5, MCF-7, HCT116, SCC4, H1299, U937, U266, MM.1S, RPMI8226 **cancer cells**	(98)
TNF-β**/**NF-κB	Calebin A acted as anti-inflammatory agent *via* suppressing TNF-β-induced NF-κB cascade in cancer cells.	*in vitro*	HCT116, HCT116R, RKO, SW480 **CRC cells**	(14, 85, 100–102)
RANKL**/**NF-κB	Calebin A affected cancer- and non-cancer-induced osteoclastogenesis by inhibiting IκBα phosphorylation, thus suppressing NF-κB activation.	*in vitro*	RAW264.7 mouse **macrophage cells**	(19)
Scleraxis**/**NF-κB	Calebin A modulated the functional linkage between NF-κB and scleraxis, down-regulated activation of IκBα and IκB-kinase.	*in vitro*	canine **tenocytes**	(17)

**Figure 3:**
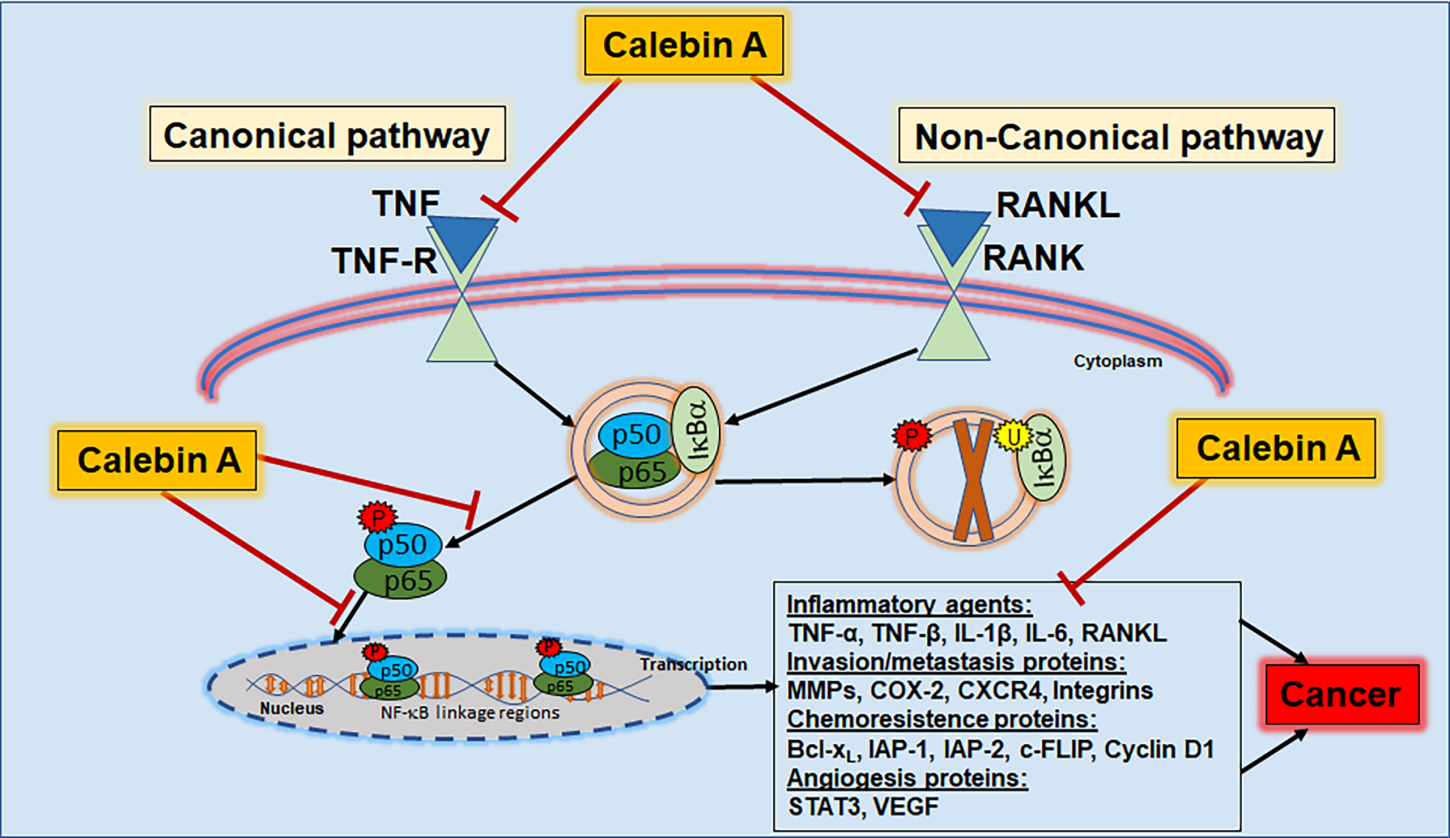
Calebin A as anti-inflammatory pathway modulator. Calebin A modulates the NF-κB cascade *via* both the canonical and non-canonical pathways and can therefore be useful in the prevention or co-treatment of inflammation, chronic diseases or cancers. TNF, tumor necrosis factor; TNF-R, tumor necrosis factor receptor; RANK, receptor activator of NF-κB; RANKL, receptor activator of NF-κB ligand; IκB, inhibitor nuclear factor of kappa light poly-peptide gene enhancer in B-cells; NF-κB, nuclear factor kappa-light-chain-enhancer of activated B-cells; IL, interleukin; MMP, matrix metalloproteinase; COX-2, cyclooxygenase-2; CXCR4, C-X-C chemokine receptor type 4; Bcl-xL, B-cell lymphoma-extra-large; IAP - inhibitor of apoptosis protein; STAT3, signal transducer and activator of transcription 3; VEGF, vascular endothelial growth factor.

For NF-κB cascade initiation, numerous signaling pathways can be involved and we will present examples for canonical and non-canonical signaling pathways that are (**Figure 3**) known up to date to provide various possibilities for Calebin A to act as an anti-inflammatory agent.

The canonical signaling pathway can be initiated with ligands binding to cell receptors, including cytokines such as TNFs to TNF-Rs. Ligand-receptor binding consequently results in the phosphorylation of IKK-inhibitors, so that IKK can phosphorylate IkBα, leading to NF-κB release and its nuclear translocation, finally resulting in target gene transcription. This process can also be initiated *via* the non-canonical pathway by RANKL binding as a ligand to the receptor activator of NF-κB (RANK) receptor. However, in contrast to the canonical pathway that includes the kinase β, the non-canonical pathway is associated with IkBα kinase α. After nuclear translocation, NF-κB is phosphorylated, activated, and induces the expression of several transcription factors as well as of inflammatory genes and cytokines such as TNF-α, TNF-β, MMPs and COX-2 (103). TNFs are cytokine signaling molecules that lead to nonspecific immune responses through the release of acute-phase proteins in response to inflammation or tissue damage (104). Furthermore, inflammation-related increased cytokine levels entail expression of the enzyme COX-2, which has a pro-inflammatory effect on the arachidonic acid metabolism (105). MMPs, on the other hand, are proteinases involved in extracellular matrix remodeling and are physiologically necessary for growth processes, what can be pathologically exploited by tumors (106). Alone or in combination, the described signaling molecules create an inflammatory response throughout the whole organism that holds risk to become a chronic condition predestined for cancer development (**Figure 3**).

Calebin A has already been shown to act as a potent anti-inflammatory agent in a variety of ways. In a comprehensive *in vitro* investigation, it was able to suppress the TNF-α-induced canonical NF-κB activation by inhibiting IκBα degradation and NF-κB binding to DNA (98) in KBM-5 (leukemia), MCF-7 (BC), SCC4 (head and neck cancer), H1299 (LC), U937 (lymphoma), U266, RPMI8226 and MM.1S (myeloma) as well as HCT116 (CRC) cells (**Table 1**). CRC cells are among the most well studied cancer cell types, which were found to exhibit cross-talk with other cell types such as fibroblasts and lymphocytes in an inflammatory tumor microenvironment, finally resulting in the release of inflammation-promoting cytokines leading to the increase of phosphorylated NF-κB in CRC cells (100). Also, TNF-β induced activation of nuclear translocation of NF-κB leads to enhanced survival and migration in four different CRC cell types (HCT116, HCT116R, RKO, SW480), but Calebin A was able to block this signaling cascade (14) (**Table 1**). Furthermore, it is known that tumor cells make use of the non-canonical pathway for NF-κB activation, because BC (MDA-MB-231) and myeloma (U266) cells have been shown to induce RANKL-mediated osteoclastogenesis in mouse macrophage (RAW264.7) cells together with increased expression and phosphorylation and degradation of the IkB and thus with the inhibition of NF-κB activation. In addition, the authors found that an inhibitory peptide specific for NF-κB delayed RANKL-promoted osteoclastogenesis, confirming that NF-κB signaling is required for the induction of RANKL-promoted osteoclastogenesis and that Calebin A therefore strongly attenuates RANKL- and RANKL-stimulated osteoclast formation by inhibiting NF-κB signaling (19) (**Table 1**).

Moreover, as another example for Calebin A’s impact on NF-κB-triggered inflammation, the correlation of up-regulated NF-κB with concomitant down-regulated expression of the transcription factor scleraxis found in canine tenocytes was reversed by Calebin A treatment. With the ability to synergistically down-regulate pro-inflammatory NF-κB and to up-regulate tendon specific scleraxis, Calebin A efficiently reduced the inflammation-mediated response in healthy cells (**Table 1**) [16]. On another note, Calebin A has been observed as a non-selective and direct inhibitor of both COX-1 (consecutive expressed) as well as COX-2 (inflammation-induced) in several independent studies (**Table 1**) (89, 99). The pro-inflammatory enzyme COX-2 is known as one of many NF-κB dependent gene end products that is involved in the most inflammatory processes and, therefore represents a major target of many classic anti-inflammatory drugs (107), further underlining the great potential of Calebin A in cancer therapy. Especially, the multiple points of attack of Calebin A in inflammatory conditions, namely the prophylactic treatment to prevent inflammation and cancer, the alternative treatment to suppress inflammatory mediators and pathways or the co-treatment strategy after cancer development, demonstrate its great potential in the therapy of chronic diseases and cancer.

Overall, Calebin A can be considered a multifunctional anti-inflammatory agent so far (**Table 1**), worthy for further investigation as its inflammation-modulatory properties may significantly contribute to the control of inflammation, not only in cancer, but also in the context of sports injuries, infectious diseases and other chronic disorders. The anti-cancer effects of Calebin A will be pointed out in the next chapter more specifically.

### 4.2 Anti-cancer effects of Calebin A

Cancer diseases, their prevention and treatment are of great importance for human health worldwide and because classical therapies are insufficient in many cases, the integration of natural, secondary plant metabolites into modern therapy conception is being scientifically explored. Therefore, in this chapter, we will summarize the currently known anti-cancer effects of the plant-derived polyphenol Calebin A (**Table 2**) in ten different cancer types overall and outline its potential role in the treatment of various cancer cell lines by modulating different signaling pathways.

**Table 2:** Calebin A as anti-cancer agent.

Cancer type	Experimental setting		Anti-cancer properties	Involved pathways	Reference
**Breast cancer**	*in vitro*	BC cells: **MCF-7**	Calebin A suppressed inflammation.	TNF-α, NF-κB	(98)
	*in vitro*	BC cells: **MDA-MB-231**	Calebin A inhibits BC cell induced osteoclastogenesis.	RANKL, IκBα, NF-κB	(19)
**Colorectal cancer**	*in vitro*	CRC cells: **HCT116**	Calebin A reduced inflammation and proliferation.	TNF-α, NF-κB	(98)
	*in vitro*	CRC cells: **HCT116, RKO, SW480**	Calebin A inhibited colonosphere formation, proliferation, invasion/migration and enhanced apoptosis.	TNF-β, NF-κB, MMP-9, CXCR4, β1-integrin, Ki-67, caspase-3	(14)
	*in vitro*	CRC cells: **HCT116** and 5-FU resistant CRC cells: **HCT116R**	Calebin A reduced survival capacity, invasion, promotes apoptosis and chemosensitized resistant CRC cells to 5-FU.	TNF-β, IKK/NF-κB, MMP-9, CXCR4, β1-integrin, Ki-67, caspase-3	(101)
	*in vitro*	CRC cells: **HCT116**	Calebin A suppressed DNA interaction, proliferation, colony formation, invasion and promoted morphological apoptotic changes.	NF-κB, Bcl-2, Bcl-x_L_, survivin, cyclin D1, MMP-9, CXCR4, caspase-3	(102)
	*in vitro*	CRC cells: **HCT116, RKO**	Calebin A blocked cell viability, proliferation, migration/invasion, EMT and increased apoptosis.	TNF-β, NF-κB, FAK, E-cadherin, vimentin, slug, smad-2, caspase-3	(85)
	*in vitro*	CRC cells: **HCT116**	Calebin A down-regulated cell vitality, colony formation, proliferation, migration, CSC activation and up-regulated apoptosis.	NF-κB, MMP-9, CXCR4, β1-integrin, Ki-67, caspase-3, ALDH1, CD44, CD133	(100)
	*in vitro*	CRC cells: **SW480**	Calebin A significantly inhibited cell growth, proliferation and viability, measured by MTT assay.	(not investigated)	(108)
**Gastric cancer**	*in vitro*	vincristine resistant GC cells: **SGC7901**	Calebin A overcame resistance, reduced cell growth, S-/G2/M-phase arrest, induced apoptosis and enhanced cytotoxicity of vincristine.	MAPK, JNK, ERK, p38, G2/M-phase, caspase-3/-8/-9, P-glycoprotein, p53, Bax	(109)
**Head/neck cancer**	*in vitro*	SCC cells: **SCC4**	Calebin A suppressed proliferation and inflammation.	TNF-α, NF-κB	(98)
**Hepatic cancer**	*in vitro*	adriamycin resistant hepatoma cells: **HepG2**	Calebin A broke down resistance and decreased the survival rate of resistant cells.	P-glycoprotein, p53, Bax, caspase-3	(109)
**Leukemia**	*in vitro*	leukemic cells: **KBM-5**	Calebin A reduced survival, inflammation, proliferation, invasion/metastasis, induced apoptosis and increased therapeutic potential of thalidomide and 5-FU.	TNF-α, NF-κB, Bcl-2, c-IAP-1, cFLIP, XIAP, cyclin D1, c-Myc, COX-2, ICAM-1, VEGF	(98)
**Lung cancer**	*in vitro*	LC cells: **H1299**	Calebin A inhibited inflammation.	TNF-α, NF-κB	(98)
	*in vitro*	cisplatin resistant LC cells: **A549**	Calebin A overcame resistance and lowered the survival rate of resistant cells.	P-glycoprotein, p53, Bax, caspase-3	(109)
**Lymphoma**	*in vitro*	lymphoma cells: **U937**	Calebin A down-regulated inflammation.	TNF-α, NF-κB	(98)
	*in vitro*	lymphoma cells: **Sup-T1**	Calebin A reduced growth and viability.	HDAC, HAT, PCAF, CYP2C9, CYP3A4, lipoxygenase	(99)
**Multiple Myeloma**	*in vitro*	myeloma cells: **U266, MM.1S, RPMI8226**	Calebin A suppressed inflammation and proliferation.	TNF-α, NF-κB	(98)
	*in vitro*	myeloma cells: **U266**	Calebin A inhibits myeloma cell induced osteoclastogenesis.	RANKL, IκBα, NF-κB	(19)
**Nerve sheath tumor**	*in vitro*	MPNST cells: **STS26T, ST8814, T265, S462TY**	Calebin A decreased cell viability, proliferation, G2/M-phase arrest and modified histones.	Akt, ERK1/2, survivin, hTERT, acetyl H3, HAT	(86)
	*in vivo*	**male Balb/c nude mice**	Calebin A reduced significant tumor size of xenograft tumors after two weeks of treatment with 100mg/kg Calebin A alone or in combination with 2mg/kg selumetinib (MEK-inhibitor).	RAS, MEK, ERK	(86)

#### 4.2.1 Breast cancer regulation by Calebin A

One of the most common forms of cancer in the world is BC, with over 2 million new cases diagnosed each year. While men are rarely affected, one of four women suffering from cancer is diagnosed with BC. While up to 10% of BC cases are genetic and hereditary, a large proportion of these diseases are caused by epigenetic alterations and hormonal changes (1). Due to the multifaceted nature of BC disease, solutions are strongly being sought for modulation of underlying triggers such as inflammation. Therefore, the application of phytochemicals showing powerful anti-inflammatory effect may be helpful here. In this context, the curcuminoid curcumin is currently being already used in clinical studies and extensive analyses of blood, urine as well as breast tissue samples, which showed clearly the strong potential of curcumin to help fighting BC, if it was consumed regularly (110). These results encourage to also further investigate the potential anti-BC properties of Calebin A. In fact, a first study (**Table 2**) showed a dose-dependent inflammation- and thus proliferation-reduction in MCF-7 (BC cells) *via* suppression of TNF-α-induced NF-κB activation (98) by Calebin A. Furthermore, in another BC cell line, MDA-MB-231 the induction of osteoclastogenesis in mouse macrophage cells (RAW264.7) was observed, supporting the idea of bone loss as major secondary BC disease. With Calebin A administration, remarkable down-regulation of the BC-induced osteoclastogenesis could be determined, whereas RANKL-associated and IκBα-mediated NF-κB signaling was clearly modulated (19), underlining Calebin A’s wide-ranging usability. Altogether, these results are of great value and the anti-inflammatory, anti-osteoporosis and anti-tumor effects of Calebin A that were found in relation to BC, should be further investigated.

#### 4.2.2 Colorectal cancer regulation by Calebin A

With an incidence of approximately 1.9 million people per year, CRC is one of the most common cancers in men and women and represents the third most frequent cancer disease worldwide (1). Research of its genesis and therapy is of great interest, so that anti-CRC effectiveness of curcuminoids such as curcumin has long been re-searched in both, *in vitro* and *in vivo* experiments (102). Also, CRC-modulatory capabilities of Calebin A have already been explored in various CRC cells such as HCT116, RKO, SW480 and HCT116R, a CRC cell line showing resistance to the chemotherapeutic agent 5-FU, as summarized in **Table 2**. Related to impeding the spread of cancer, the prevention or control of inflammation are of central importance. Therefore, the ability of Calebin A to down-regulate the major inflammatory transcription factor NF-κB and its genetic end products in HCT116 cells, both when NF-κB was TNF-α or TNF-β induced, seemed very promising for further research. In addition, CRC cell vitality, colony formation and proliferation were also found to be inhibited by the down-regulation of tumor-promoting biomarkers such as the surface protein and cell adhesion molecule β1-integrin, cell division protein Ki-67 and cyclin D1, a regulator implicated in the cell cycle (98, 101, 108). Furthermore, epithelial-mesenchymal transition (EMT), attached to a change of cancer cell morphology from epithelial to mesenchymal shape (111) and thus metastatic activity and resulting invasion were inhibited in Calebin A treated HCT116, RKO and SW480 cells (**Table 2**). This inhibition was found to be associated with a high E-cadherin expression functioning as an epithelial marker and, at the same time, down-regulation of the mesenchymal marker vimentin, main EMT-related transcription factor slug, metastasis-parameter CXCR4 and the invasion-parameter MMP-9 (14, 85). Moreover, Calebin A suppressed cancer stem cell (CSC) activation by down-regulation of cluster of differentiation (CD)133, CD44 and aldehyde dehydrogenase (ALDH)1 and promoted apoptosis *via* inhibition of anti-apoptotic Bcl-2, B-cell lymphoma-extra-large (Bcl-xL) as well as survivin and activation of pro-apoptotic caspase-3 (100, 102). Beyond the remarkable results in HCT116, 5-FU resistant HCT116R cells were also found to be restricted in their survival and invasion ability by Calebin A treatment and additionally the appearance of typical apoptotic bodies was observed. Interestingly, Calebin A succeeded in overcoming the resistance by modulating signaling cascades and the HCT116R cells were markedly chemosensitized by Calebin A (101), too (**Table 2**). Overall, these promising results should be studied in *in vivo* experiments in a next step in order to verify the anti-cancer capacity of Calebin A, as it has already been done with divers curcuminoids. For example, curcumin has already been used in a clinical trial (112) in patients with an advanced stage of CRC and after 8 weeks of consumption of curcuminoids capsules (500 mg/day), their serum levels of inflammation were markedly reduced compared to the placebo group, leading to an improved quality of life. All things considered; these profound results are encouraging to expand further cancer research related to the application of Calebin A.

#### 4.2.3 Gastric cancer regulation by Calebin A

Gastric cancer (GC) is an also very commonly appearing cancer disease, with over 1 million new cases per year globally, whereas it affects men significantly more often than women and infections with the gram-negative rod bacterium *Helicobacter pylori*, associated with chronic inflammations, are considered as main risk factor (1). As the resistance to common anti-tumor drugs represents a great challenge in this particularly aggressive cancer, the search for new co-treatment options, also by using phyto-pharmaceuticals, continues. With focusing this issue, an *in vitro* examination (**Table 2**) was conducted with SGC7901 cells, which showed resistance against the chemotherapeutic drug vincristine. During the investigations, Calebin A was found to inhibit the growth of the chemoresistant GC cells by decreasing them in the cell cycle S-phase, leading to a G2/M-phase arrest. Additionally, Calebin A dose-dependently increased the activity of pro-apoptotic caspases-3, -8 and -9 as well as mitogen-activated protein kinase p38 and, at the same time, decreased the main family members of MAPK, JNK and ERK, thus improved apoptotic activity. To be more particular, Calebin A was able to act as a reversal agent of anti-cancer drug resistance by mediating the membrane P-glycoprotein. Therefore, treatment of GC cells with Calebin A did overcome the vincristine resistance, thus dose-dependently enhanced cytotoxicity (109) of this chemotherapeutic remedy (**Table 2**). In summary, it might be worthwhile to investigate the co-treatment options of Calebin A in context of GC therapy in more detail. As the curcuminoid curcumin, which has been isolated earlier from *Curcuma* species, has already shown an inhibition of *Helicobacter pylori* induced NF-κB and IL-8 increase (113), it should also be investigated whether Calebin A could act as prophylaxis agent against stomach inflammation as well as inflammation-provoked GC.

#### 4.2.4 Head and neck cancer regulation by Calebin A

Head and neck cancer (HNC) includes malignant neoplasms of the upper aero-digestive tract, salivary glands as well as paranasal sinuses and the total global incidence is about 0.6 million (114) people every year. The respiratory tract and nearby structures are potential entry points for inflammation-causing pathogens and are therefore constantly involved in inflammation defense. As in all parts of the body, acute inflammations can turn into chronic infections and, which can consequently lead to the development of cancer (7). Based on this, it seems reasonable in terms of prevention to include natural polyphenols in the inflammation defense and a primary study (**Table 2**) showed the ability of Calebin A to suppress inflammation and proliferation in a dose-dependent manner *via* inhibiting TNF-α-induced NF-κB activation (98) in HNC cells (SCC4). With this result, a basic framework has been established for the initial investigation of further anti-inflammatory and anti-HNC mechanisms of Calebin A on different HNC cell lines *in vitro*.

#### 4.2.5 Hepatic cancer regulation by Calebin A

The incidence of hepatic cancer (HC), which includes approximately 80% of hepatocellular carcinomas, up to 15% intrahepatic cholangiocarcinomas as well as a few rare forms, accounts for about 0.84 million of people worldwide, while the HC-related mortality counts 0.78 million patients annually (1), while these diseases affect more men than women. In order to preserve the liver as a central metabolic organ, a new approach has been made to supplement classical chemotherapy concomitantly with plant substances such as curcumin (115). As safe polyphenols showed great potential here, the effect of Calebin A on HepG2 cells was investigated as well (**Table 2**). Noteworthy, co-treatment with Calebin A and adriamycin could significantly re-duce the survival of HC cells compared to treatment with chemotherapeutic agent adriamycin alone, even in HepG2 cells, which are resistant against adriamycin. To achieve these results, Calebin A modulated P-glycoprotein and induced apoptosis *via* p53, Bcl-2-associated X protein (Bax) and caspase-3 activation (109). The main risk factors for HC include infections, for example such of viral origin like HBV or HCV (116). With regard to the already known anti-inflammatory effects of Calebin A, a next conceivable step could be to investigate whether Calebin A is able to prevent the development of liver inflammation or to fight it in order to prevent the chronification, thus the degeneration of hepatic cells, leading to cancer.

#### 4.2.6 Leukemia regulation by Calebin A

Leukemia as umbrella term, summarizes a group of cancers, including acute or chronic and lymphoid or myeloid progressions. Taken together, they have a total worldwide incidence rate of almost 0.48 million patients per year (117). Due to the variability of the pathogenesis, different and above all individual therapy concepts are necessary for efficient leukemia therapy. Natural polyphenols could be a particularly useful approach to support traditional treatment here, as they do not have a mono-centric effect but act as multi-targeting agents intervening in different molecular processes of disease by modulating various signaling pathways. In KBM-5 leukemic cells, representing chronic myeloid type of leukemia, Calebin A has been demonstrated to exhibit various multi-targeting anti-cancer effects as described in **Table 2**. Firstly, it could prevent TNF-α-associated NF-κB binding to DNA by the suppression of IkBα-degradation and further activation of inflammation-specific tissue enzyme COX-2. Thereby Calebin A caused a broad anti-inflammatory effect. Moreover, Calebin A was able to suppress TNF-α/NF-κB-related gene end products, involved in proliferation, metastasis and invasion, such as cell cycle regulator cyclin D1, protooncogene c-Myc, intercellular adhesion molecule (ICAM) 1 and VEGF. Furthermore, apoptosis was found to be up-regulated *via* inhibition of anti-apoptotic proteins like x-linked inhibitor of apoptosis protein (XIAP), Bcl-2, c-IAP-1 and cFLIP. The increased apoptotic activity even enabled a Calebin A-dependent increase in the therapeutic efficiency of classic chemotherapeutic drugs, thalidomide and 5-FU (98), in mentioned leukemia (KBM-5) cells. The versatile results of this study are promising and in accordance to earlier findings, proving that curcumin as another turmeric compound suppressed TNF-related NF-κB activation as well as proliferation (118) in leukemia cells. Overall, the investigation of Calebin A’s anti-leukemic effect on cells of other leukemia types *in vitro* would be of great relevance in the search of novel treatment strategies.

#### 4.2.7 Lung cancer regulation by Calebin A

LC represents a large proportion of patients with over 2 million new cases and moreover, about 1.8 million LC-related patient deaths per year. More than 80% of LC in Western populations are associated with tobacco smoking (1), leading to inflammation *via* IL-8 and p38/MAPK signaling cascades (119), among others. A blockage of pro-inflammatory TNF-α pathway, for example, has been shown to prevent the establishment of chronic lung inflammation (120), relevant as chronic inflammations represent a high risk to develop LC. Consistent with the knowledge that natural polyphenols are potent inflammation modulators, Calebin A dose-dependently showed a reduced proliferation in human lung adenocarcinoma cells (H1299) by inhibiting TNF-α-induced NF-κB activation (**Table 2**) and thereby a suppression of inflammation (98). In treatment-intense diseases such as LC, the rapid development of resistances against classical chemotherapeutic agents also plays a decisive role. Therefore, it is a promising finding, that Calebin A significantly reduced the survival of cisplatin resistant A549 LC cells by modulating P-glycoprotein as well as pro-apoptotic agents p53, Bax and caspase-3 (109), evidencing that Calebin A was able to overcome resistance of these cancer cells *in vitro* (**Table 2**). Collectively, further expansion of research in this area is necessary and Calebin A represents a promising operator in fighting LC-related inflammation and even in overcoming chemoresistance in the future.

#### 4.2.8 Lymphoma regulation by Calebin A

The total number of over 0.5 million new non-hodgkin lymphoma und nearly 83.000 new hodgkin lymphoma diagnoses (121) every year, ensures a high global number of patients with a malignant lymphoma, whereas all types of lymphoid cells can be affected. Both, organs and cells of the lymphatic system are of central im-portance in the immune defense of humans and therefore inevitable constantly confronted with the prevention or fight against inflammations. Underlining this, histiocytic lymphoma cells (U937) showed increased activation of the inflammatory NF-κB cascade activated by TNF signals (122), representing a signaling pathway that acts as a target of natural polyphenolic agents such as Calebin A. As shown in **Table 2**, Calebin A inhibited NF-κB-promoted inflammation by intervening in the pathological TNF-α/NF-κB activation in U937 lymphoma cells (98). Additionally, Calebin A acted anti-oxidative, anti-inflammatory and reduced growth as well as viability of Sup-T1 lymphoblastic lymphoma cells by down-regulation of COX-1, NF-κB-related COX-2, lipoxygenase, cytochrome (CYP)2C9 and CYP3A4 enzymes. Moreover, in the same study (**Table 2**), Calebin A inhibited histone acetyltransferase (HAT), histone deacetylase (HDAC) and P300/CBP-associated factor (PCAF) (99), which are involved in histone acetylation and thus epigenetic modification, thus playing a central role in cancer development. In summary, epigenetic mutations are demonstrably associated with lymphoma development (123), leading to the assumption that Calebin A, among curcuminoids, provides multiple lymphoma-inhibiting effects *via* modulation of inflammation and epigenetic processes.

#### 4.2.9 Multiple myeloma regulation by Calebin A

With 0.18 million new cases worldwide per year (124), multiple myeloma (MM) belongs to a smaller group of cancers, but represents the most frequent malignant neoplasm of the bone marrow. MM is also known as plasma cell myeloma, which illustrates the link to immune and inflammatory processes. After it has been already known that inflamed cells of MM are sensitive to curcuminoids (125), MM cell lines (U266, RPMI8226, MM.1S) were treated with Calebin A in a separate study listed in **Table 2**. Interestingly, Calebin A presented as dose-dependent suppressor of inflammation and proliferation *via* inhibiting TNF-α-induced NF-κB activation (98) in all three investigated cell lines. Moreover, as shown in **Table 2**, U266 myeloma cells were able to induce osteoclastogenesis in RAW264.7 mouse macrophage cells, reinforcing bone loss as relevant secondary cancer disease in MM. Noteworthy, Calebin A down-regulated this cancer-induced osteoclastogenesis by modulation of RANKL/IκBα axis and thereby NF-κB phosphorylation (19). Overall, these results underline Calebin A’s wide-ranging scope and the usefulness of further investigations on its anti-carcinogenic effects.

#### 4.2.10 Nerve sheath tumor regulation by Calebin A

Malignant peripheral nerve sheath tumors (MPNST) are rare diseases with a low lifetime prevalence of 0.001% (126), but there is a strong association with neurofibromatosis type 1, a common genetic neurological disease, represented by 50% of patients with a MPNST being also affected by neurofibromatosis type 1 (127). As the courses of rare diseases are often difficult to study, researchers are searching for a suitable extension of therapy, for example through natural polyphenols such as curcumin, which has been shown to induce reactive oxygen species in neurofibromatosis type 1-associated MPNST cells (128). Therefore, a combined *in vitro*/*in vivo* investigation (**Table 2**) has addressed Calebin A’s modulatory capabilities against MPNST. In four different MPNST cell lines (STS26T, ST8814, T265, S462TY), Calebin A treatment markedly and dose-dependently reduced the vitality of the tumor cells, thus their proliferation capacity, too. A disclosure of the mechanisms of action revealed an intervention in the cell cycle, leading to a decreased cell population at G2/M phase by the phytopharmaceutical Calebin A. Furthermore, a reduction of Akt, acetylated histone H3, phosphorylated ERK1/2 as well as survivin and telomerase reverse transcriptase (hTERT) protein level, related to tumorigenesis, were observed after treatment with Calebin A. Interestingly, Calebin A showed the ability to modify histones by inhibiting the promoters in hTERT and BIRC5 genes what is of great relevance considering as the activity of HAT and HDAC are key players in the epigenetic transcription control. Indeed, the enzymatic action of HAT was clearly impaired, but not that of HDAC. At length, this inhibition resulted in a significant reduction in the proliferation rate of MPNST cells. These results were further supported by a significant Calebin A-induced (100 mg/kg) tumor size reduction in a xenograft mouse model within two weeks (**Table 2**), regardless of whether Calebin A was administered alone or in combination with selumetinib (2 mg/kg), a MEK inhibitor (86). Altogether, because epigenetic mechanisms are crucial for different clinical outcomes of MPNST patients (129), the results of this study are promising and hold great potential for deeper investigation.

#### 4.2.11 Bottom line of Calebin A’s cancer regulation

**Figure 4:**
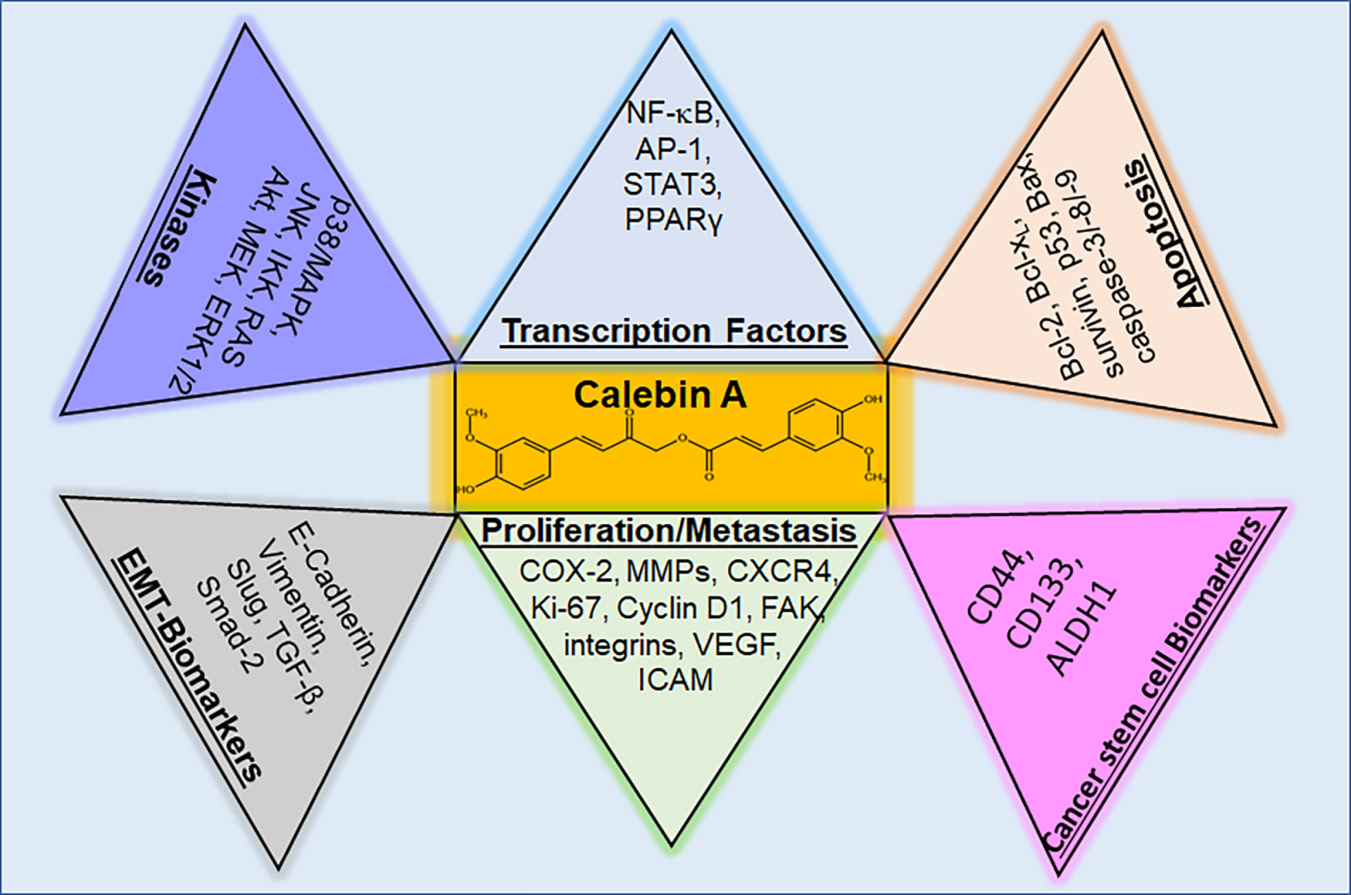
Regulatory molecular targets of Calebin A in cancer cells. Calebin A, as a multifunctional polyphenol, has the ability to modulate kinases and transcription factors, but also parameters for cancer stem cells, EMT, proliferation, metastasis and apoptosis in cells from different types of cancer. p38/MAPK, protein kinase of 38 kDa/mitogen-activated protein kinase; JNK, c-Jun N-terminal kinase; IKK, IκB kinase; RAS, rat sarcoma; Akt, serine-threonine protein kinase B; MEK, mitogen-activated protein kinase kinase; ERK 1/2, extracellular signal-regulated kinase; NF-κB, nuclear factor kappa-light-chain-enhancer of activated B-cells; AP-1, adaptor protein-1; STAT3, signal transducer and activator of transcription 3; PPARγ, peroxisome proliferator-activated receptor γ; Bcl-2, B-cell lymphoma 2; Bcl-xL, B-cell lymphoma-extra-large; Bax, Bcl-2-associated X protein; CD, cluster of differentiation; ALDH, aldehyde dehydrogenase; COX-2, cyclooxygenase-2; MMP-9, matrix metalloproteinase-9; CXCR4, C-X-C chemokine receptor type 4; FAK, focal adhesion kinase; VEGF, vascular endothelial growth factor; ICAM, intercellular adhesion molecule; TGF, tumor growth factor.

Overall, Calebin A is an effective anti-inflammatory (**Table 1**) and anti-cancer agent (**Table 2**), possessing the ability to regulate various molecular targets in cancer cells (**Figure 4**), worth for further exploration as a preventive resource or a coupling drug in cancer therapy for instance. As the currently known mechanisms of action are mainly based on *in vitro* studies so far, *in vivo* studies for verification of the transferability to living organisms and ultimately to cancer patients should be pursued in the future.

## 5 Future area of research

**Figure 5:**
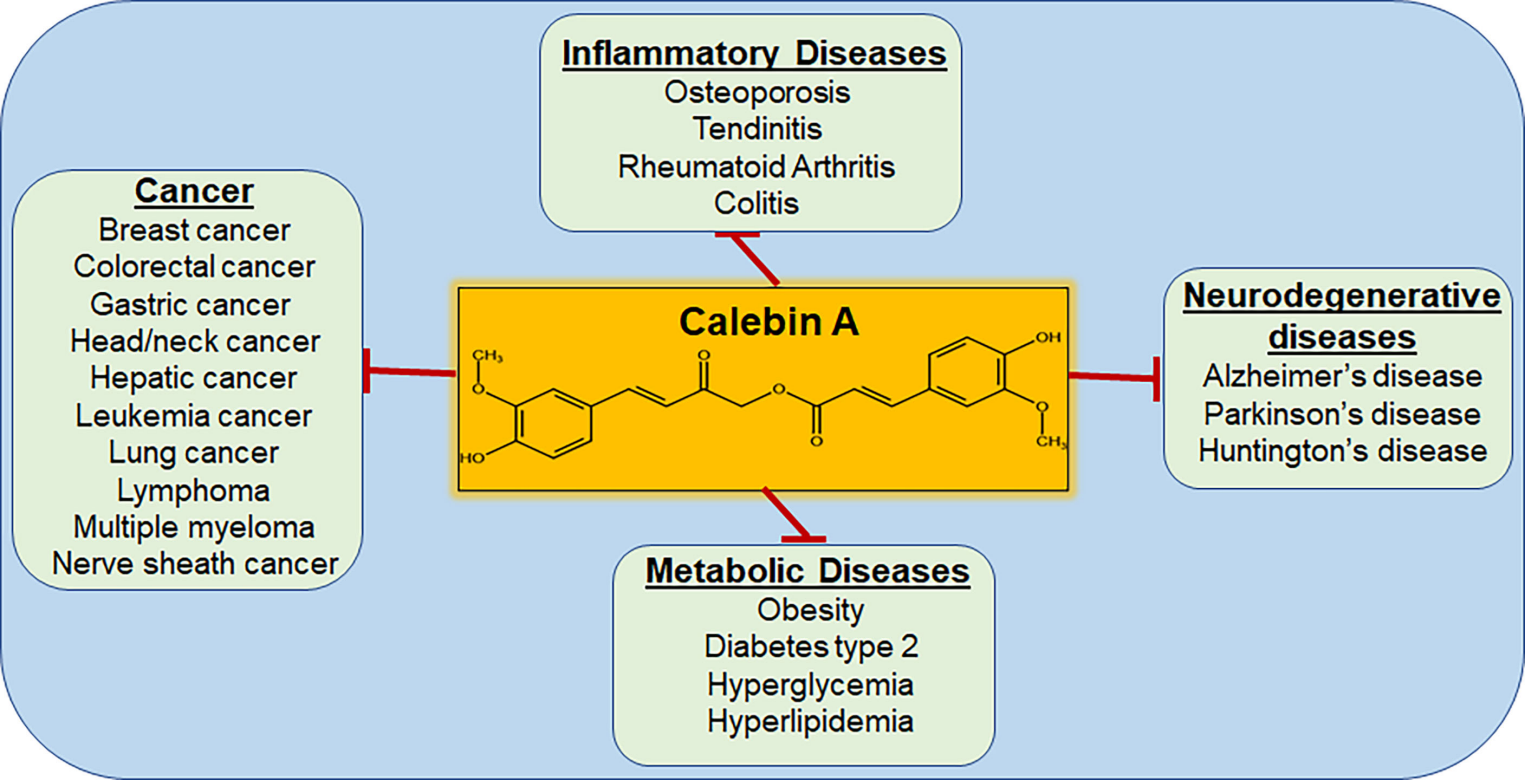
Diseases that are targeted by Calebin A. As a multi-target molecule, Calebin A may play a regulatory role in various diseases, including cancer.

Research of the health-promoting effects of turmeric is almost as old as the internationally documented publications, so it was proposed as a remedy against gonorrhoea (130) as early as 1876. After curcumin, which has a content of 2-5% in the turmeric root (88), was isolated, science initially focused on its precise examination. The discovery of curcumin’s anti-bacterial potential in 1949 (131) was followed by its anti-oxidative activity in 1976 (132) and finally in 1985, the exploration of its anti-cancer properties (133) started. Only 20 years ago, in 2002, Calebin A was isolated (18) as further turmeric compound. Within this time, enormous insights were already gained confirming the presumed health-promoting power (**Figure 5**) of Calebin A and focusing on combating inflammation by modulation of diverse steps of NF-κB pathway and inflammation-based diseases such as cancers (17, 86). In this respect, the histone modulating ability of Calebin A (99) is particularly interesting, as a large proportion of tumors appear to be based on epigenetic changes (134). For the rapid progress of relevant insights, the preliminary work done in curcumin-research was certainly a valuable guideline, and so it can be assumed that Calebin A will show great potential for its usability in many diseases in the near future, too. This would be of great benefit since inflammation is still one of the main causes responsible for many of people’s health problems (7). Any further treatment option holds the potential of help in chronic disease therapy (**Figure 5**) and Calebin A has already been proven to provide the multi-modulatory ability to protect healthy cells on the one hand, and to eliminate pathological cells on the other hand. Taken together, various studies investigating on the effects of Calebin A on different types of tumors and thus its impact on major inflammatory signaling pathways like NF-κB, show that Calebin A as a new compound of turmeric reveals not only anti-oxidative and anti-inflammatory but especially also anti-tumor effects, holding great potential as a phytopharmaceutical agent in the treatment of diseases. Therefore, Calebin A’s modulating capacity of diverse signaling pathways involved in chronic pathologies encourages for further investment of cross-disease research of Calebin A in therapeutic applications as a co-treatment or coupling molecule for classic medication as well as for prophylaxis purposes against inflammation-associated diseases and cancer.

## Data availability statement

The original contributions presented in the study are included in the article/**Supplementary Material**. Further inquiries can be directed to the corresponding author.

## Author contributions

AB, A-LM, and MS designed this study. AB and A-LM wrote the manuscript. BA and AK reviewed the manuscript. MS created the figures and supervised the preparation of the manuscript. All authors have read and agreed to the published version of the manuscript.

## Acknowledgments

We thank Andreas Eimannsberger for excellent technical assistance. We note, that the research was conducted as a part of the doctoral thesis of Aranka Brockmueller to be submitted to Fachbereich Humanmedizin, Ludwig-Maximilians-University Munich, Germany.

## Conflict of interest

The authors declare that the research was conducted in the absence of any commercial or financial relationships that could be construed as a potential conflict of interest.

## Publisher’s note

All claims expressed in this article are solely those of the authors and do not necessarily represent those of their affiliated organizations, or those of the publisher, the editors and the reviewers. Any product that may be evaluated in this article, or claim that may be made by its manufacturer, is not guaranteed or endorsed by the publisher.

## Abbreviations

5-FU- fluorouracilacetyl H3- acetylated histone 3, Akt- serine-threonine protein kinase B, ALDH - aldehyde dehydrogenase, AMPK - AMP-activated protein kinase, AP- adaptor protein, ATF- activating transcription factor, Bax- Bcl-2-associated X protein, BC- breast cancer, Bcl-2- B-cell lymphoma 2, Bcl-xL- B-cell lymphoma-extra large, BIRC5- surviving, CD- cluster of differentiation, C/EBP - CCAAT/enhancer-binding protein, COX- cyclooxygenase, CRC- colorectal cancer, CSC- cancer stem cell, CXCR4- C-X-C chemokine receptor type 4, CYP- cytochrome, DNA- deoxyribonucleic acid, DPP-IV- dipeptidyl peptidase IV, EMT- epithelial-mesenchymal transition, ERK- extracellular signal-regulated kinase, FAK- focal adhesion kinase, FAS- fatty acid synthase, GC- gastric cancer, GPCR- G-protein coupled receptor, HAT- histone acetyltransferase, HC- hepatic cancer, HDAC - histone deacetylase, HNC- head and neck cancer, hTERT- telomerase reverse transcriptase, IAP- inhibitor of apoptosis protein, ICAM- intercellular adhesion molecule, IκB- inhibitor nuclear factor of kappa light polypeptide gene enhancer in B-cells, IKK- IκB kinase, IL- interleukin, JAK- Janus kinase, JNK- c-Jun N-terminal kinase, LC- lung cancer, MAPK- mitogen-activated protein kinase, MEK- mitogen-activated protein kinase kinase, MM- multiple myeloma, MMP- matrix metalloproteinase, MPNST- malignant peripheral nerve sheath tumor, mTOR- mammalian target of rapamycin, NF-κB- nuclear factor kappa-light-chain-enhancer of activated B-cells, p38- protein kinase of 38 kDa, PCAF- P300/CBP-associated factor, PI3K- phosphoinositide 3-kinase, PIP3- phosphatidylinositol 3,4,5-trisphosphate, PPARγ- peroxisome proliferator-activated receptor γ, RANK- receptor activator of NF-κB, RANKL- receptor activator of NF-κB ligand, RTK- receptor tyrosine kinase, SCC- squamous cell carcinoma, STAT- signal transducer and activator of transcription, TLR- Toll-like-receptor, TNF- tumor necrosis factor, TNF-R- tumor necrosis factor receptor, VEGF- vascular endothelial growth factor, XIAP- x-linked inhibitor of apoptosis protein

## Glossary

**Table:**

5-FU	fluorouracil
H3	acetylated histone 3
Akt	serine-threonine protein kinase B
ALDH	aldehyde dehydrogenase
AMPK	AMP-activated protein kinase
AP	adaptor protein
ATF	activating transcription factor
Bax	Bcl-2-associated X protein
BC	breast cancer
Bcl-2	B-cell lymphoma 2
Bcl-xL	B-cell lymphoma-extra large
BIRC5	surviving
CD	cluster of differentiation
C/EBP	CCAAT/enhancer-binding protein
COX	cyclooxygenase
CRC	colorectal cancer
CSC	cancer stem cell
CXCR4	C-X-C chemokine receptor type 4
CYP	cytochrome
DNA	deoxyribonucleic acid
DPP-IV	dipeptidyl peptidase IV
EMT	epithelial-mesenchymal transition
ERK	extracellular signal-regulated kinase
FAK	focal adhesion kinase
FAS	fatty acid synthase
GC	gastric cancer
GPCR	G-protein coupled receptor
HAT	histone acetyltransferase
HC	hepatic cancer
HDAC	histone deacetylase
HNC	head and neck cancer
hTERT	telomerase reverse transcriptase
IAP	inhibitor of apoptosis protein
ICAM	intercellular adhesion molecule
IκB	inhibitor nuclear factor of kappa light polypeptide gene enhancer in B-cells
IKK	IκB kinase
IL	interleukin
JAK	Janus kinase
JNK	c-Jun N-terminal kinase
LC	lung cancer
MAPK	mitogen-activated protein kinase
MEK	mitogen-activated protein kinase kinase
MM	multiple myeloma
MMP	matrix metalloproteinase
MPNST	malignant peripheral nerve sheath tumor
mTOR	mammalian target of rapamycin
NF-κB	nuclear factor kappa-light-chain-enhancer of activated B-cells
p38	protein kinase of 38 kDa
PCAF	P300/CBP-associated factor
PI3K	phosphoinositide 3-kinase
PIP3	phosphatidylinositol 3, 4, 5-trisphosphate
PPARγ	peroxisome proliferator-activated receptor γ
RANK	receptor activator of NF-κB
RANKL	receptor activator of NF-κB ligand
RTK	receptor tyrosine kinase
SCC	squamous cell carcinoma
STAT	signal transducer and activator of transcription
TLR	Toll-like-receptor
TNF	tumor necrosis factor
TNF-R	tumor necrosis factor receptor
VEGF	vascular endothelial growth factor
XIAP	x-linked inhibitor of apoptosis protein
