# Supplementary figures and images for: Multifunctionality of Calebin A in inflammation, chronic diseases and cancer
Source: Front Oncol. 2022 Sep 16;12:962066. doi: 10.3389/fonc.2022.962066 (PMC9523377; doi:10.3389/fonc.2022.962066)

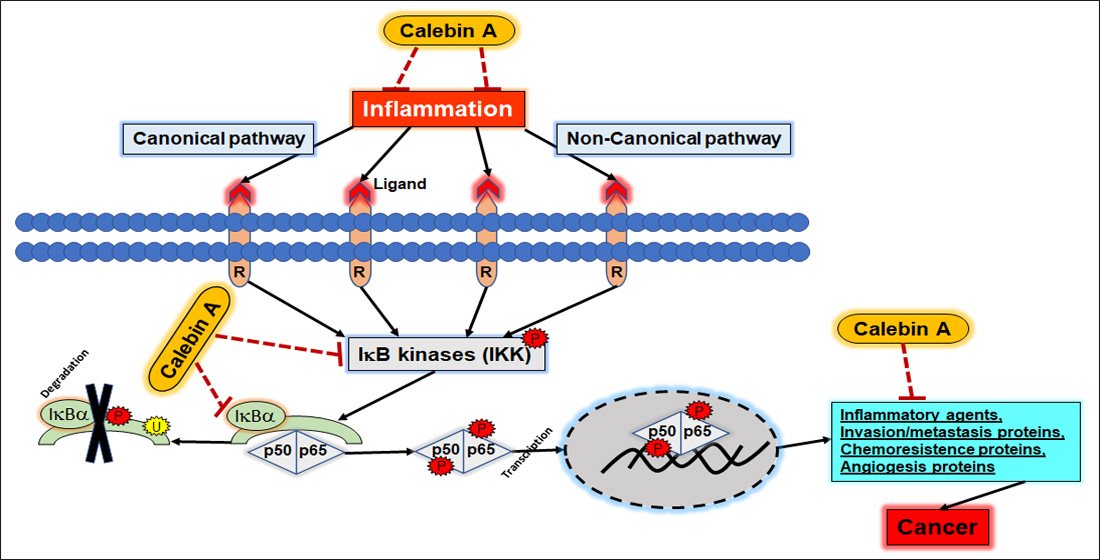

Supplement: Supplementary file 1 [file Image_1.jpeg]
